# Cytokine profiles of lupus patients with or without nephritis

**DOI:** 10.1186/ar3980

**Published:** 2012-09-27

**Authors:** BH Rovin, H Song, CL Hines, X Zhang

**Affiliations:** 1 Ohio State University, Columbus, OH, USA

## Background

It remains unclear why some patients with SLE develop lupus nephritis (LN) and others do not. A reasonable hypothesis is that the inflammatory/immunomodulatory mechanisms in patients with LN are either qualitatively different than in nonrenal (NR) lupus, or that similar pathways are activated but the magnitude of activation is greater in LN. Studies of individual or small groups of candidate cytokines support this idea. To test this hypothesis we examined baseline serum and peripheral blood mononuclear cell (PBMC) cytokine profiles of SLE patients with active LN or NR (mainly arthritis) lupus before entry into a clinical trial of Laquinimod (Teva Pharmaceuticals) as an adjunct anti-inflammatory agent for SLE. We sought to determine whether NR or LN was associated with a distinct pattern or level of cytokine expression.

## Methods

Serum and PBMC from 49 NR and 16 LN patients were collected. PBMC were cultured in medium with or without lipopolysaccharide (LPS), phytohemagglutinin (PHA), or tetanus toxoid (TT). PBMC proliferation was measured after 48 hours, and 38 cytokines were measured in the PBMC-conditioned supernatant by Luminex multiplexing. The same cytokines were measured in the sera.

## Results

LN patients were younger (37 ± 9 vs. 47 ± 14, *P *< 0.006), had a higher serum creatinine (91.6 ± 28.6 vs. 69.6 ± 16.5 μmol/l, *P *< 0.0003), higher BILAG score (13.6 ± 8.2 vs. 7.2 ± 3.2, *P *< 0.0001), higher baseline corticosteroid dose (18.3 ± 1.3.3 vs. 8 ± 8 mg/day, *P *< 0.004), and were more likely to enter the study taking a concomitant immunosuppressive agent (MMF; 50 vs. 22%). There were no quantitative differences in NR and LN serum cytokine levels for most analytes that were within the limits of detection. Eotaxin and soluble TNF receptor II were greater in LN sera (*P *= 0.037 and *P *= 0.022, respectively). Interestingly, IL-17 was not detectable in any LN sample, but was present in 17% of NR sera. LN and NR PBMC proliferated equally well to LPS and PHA, but in response to TT proliferation of NR PBMC was greater than LN PBMC (*P *< 0.0001). TT did not induce LN PBMC cytokine production. LPS and PHA induced significant increases in production of 24 of the 38 measured cytokines from PBMC, but there were few differences between LN and NR. LPS-induced IL-7 and IL-12 levels were higher from LN PBMC (*P *< 0.02), and PHA induced more IL-5 production by NR PBMC (*P *= 0.008).

## Conclusion

Unexpectedly, no cytokine signature that distinctly separates LN from NR emerged from this survey. This may be due to the greater baseline level of immunosuppression in LN patients, but even with immunosuppression the LN was very active. It is intriguing to speculate that differences, perhaps genetic, in the kidney's response to inflammation, rather than differences in circulating proinflammatory cytokines, determine susceptibility to LN in SLE patients.

